# Mixed Reality (MR) Based Slit Lamp: The First Step Toward XR-Ophthalmology

**DOI:** 10.21203/rs.3.rs-7180156/v1

**Published:** 2025-07-29

**Authors:** Rui Zhou, Wei-Chiang Lin, Byron L Lam, Rong Wen, Noble Amadi, Shuliang Jiao

**Affiliations:** 1 Department of Biomedical Engineering, Florida International University 10555 W Flagler St, Miami, FL 33174, USA; 2 Bascom Palmer Eye Institute, University of Miami Miller School of Medicine 163810 Ave, Miami, FL 33136

## Abstract

This study demonstrates the concept of Mixed Reality (MR) based slit lamp (MR-SLP), which can display real-time stereoscopic diagnostic images of the eye both locally and remotely, making it suitable for stereoscopic tele-ophthalmology applications. In MR-SLP, two high-resolution video cameras were mounted on the left and right viewing channels of a conventional slit lamp to capture corresponding diagnostic images in real time, which were then transmitted to multiple MR headsets via a broadcast network. The operator wears the MR headset to see the stereoscopic diagnostic images and operates the slit lamp using the MR’s see-through function. The system’s depth perception, a key feature inherited from the slit lamp, was quantitatively tested using a tube-threading task. The feasibility of tele-ophthalmology was evaluated across multiple sites with subjective feedback from participants. The results showed no observable differences in image quality or depth perception between local and remote sites, and ophthalmologists participated in this study reported a user experience with the MR-SLP that was comparable to a conventional slit lamp. Furthermore, the tube-threading test indicated that the time taken to complete the task was similar when using the MR-SLP compared to the direct view through the eyepieces. This study successfully demonstrates the feasibility of the MR-SLP for transmitting high-quality stereoscopic slit lamp images to multiple MR devices in real-time, without distance limitations, wherever an internet connection is available.

## Introduction

The slit lamp biomicroscope is a fundamental diagnostic tool in ophthalmology for detailed examination of both the anterior and posterior segments of the eye^1^ with stereoscopic visualization^2^. Traditional slit lamps require the operator to view through the eyepieces to obtain a stereoscopic perspective. This necessity means the operator must stay close to the patient, which has become more concerning since the COVID-19 pandemic, given close contact increases the risk of transmitting infectious diseases^3,4^. Modern slit lamps frequently come with a camera, whether dedicated or integrated into a smartphone, allowing them to capture images and videos of the eye during examinations. This feature is especially beneficial for data collection, telemedicine, and education, as captured images and videos can be viewed on a display, stored electronically, and shared remotely^5–7^. Current camera-equipped slit lamps often utilize a single-channel design, resulting in the loss of stereoscopic information and, consequently, depth perception during image and video capture. Without depth perception, it can be challenging to visualize subtle anatomical variations and perform procedures guided by the slit lamp view^8^.

Mixed Reality (MR) technology enables interactive experiences that blend both real and virtual environments by using a head-mounted display (HMD). HMD is a wearable device designed to fit comfortably over the user’s head, equipped with a stereo camera and individual screens for each eye. Additional sensors are incorporated to accurately track head and eye movements. The MR’s see-through function allows users to view the real world with depth perception. By seamlessly blending digital content with the real world, MR facilitates intuitive interaction and manipulation of virtual objects within the user’s actual environment. These advancements make MR a promising tool for applications such as 3D medical data visualization and telemedicine^9,10^.

In this paper, we present the Mixed Reality based slit lamp (MR-SLP), which integrates MR technology with a traditional slit lamp. The MR-SLP enables local operators to interact naturally with patients using its see-through feature, while simultaneously providing a detailed stereoscopic view of the examination area. Additionally, the MR-SLP can stream this stereoscopic view in real time to physicians at remote locations. When accessed via mixed reality (MR), virtual reality (VR), or augmented reality (AR) headsets, remote physicians gain an immersive perspective of the examination, enhancing their ability to participate. This functionality supports collaborative decision-making, as remote specialists can actively guide local operators throughout the examination process. Furthermore, the MR-SLP enables the recording of stereoscopic diagnostic videos and images, thereby enhancing clinical documentation and expanding educational opportunities with 3D capabilities. Given that the architecture of our system is adaptable to VR, AR, and MR platforms – collectively known as Extended Reality (XR) – we propose the term XR-Ophthalmology to cover technologies that integrate XR into ophthalmic applications.

## Results

### The MR-SLP system

**Fig. 1.**
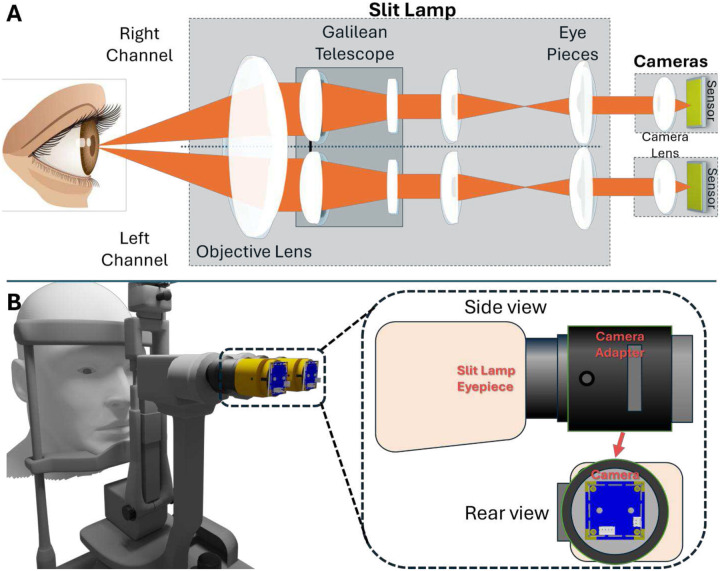
Design of the MR-SLP system. (A) Schematic of the optical path of MR-SLP. Light reflected by the examined area enters the front objective lens, then proceeds through the Galilean telescope assembly and the eyepieces before reaching the camera lens and being detected by imaging sensors. (B) Adaptation of a traditional slit lamp for MR-SLP functionality. Two cameras are mounted on the slit lamp eyepieces using adapters, allowing for stereoscopic ocular imaging.

**Fig. 2.**
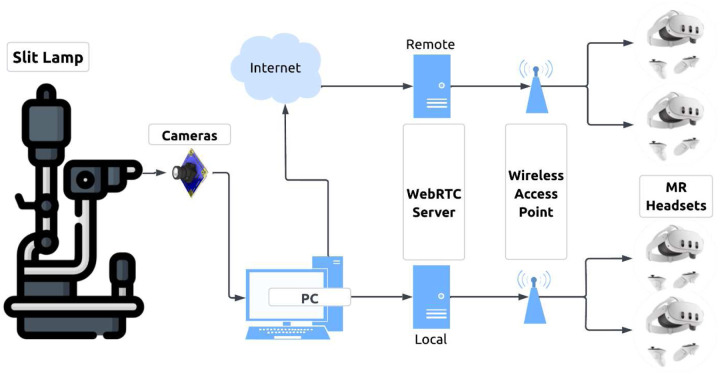
Broadcasting system architecture. The videos captured by the cameras on an MR-SLP are processed by a local PC and transmitted wirelessly to local and remote MR headsets via WebRTC servers.

The MR-SLP system was designed to stream real-time, stereoscopic diagnostic video from a slit lamp to MR headsets. Two video cameras were mounted on the left and right eyepieces of a conventional slit lamp using custom-designed adapters. A schematic of the system is shown in Fig 1. Videos captured by the two cameras were first streamed with USB connection to a local computer for processing. The processed video was synchronously transmitted to local and remote WebRTC server to broadcast wirelessly to MR headsets. The headsets then render the stream into a stereoscopic view. A diagram of the broadcasting architecture is shown in Fig. 2.

### Image Quality of MR-SLP – Spatial Resolution

**Fig. 3.**
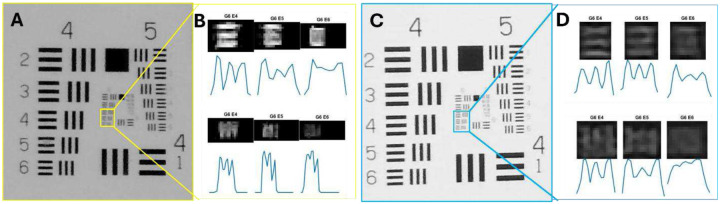
Resolution target imaging. (A) Image of the line pairs (lps) from Groups 4–6 of the resolution target captured directly by the MR-SLP camera. (B) The upper panel shows the intensity profiles across the horizontal lps of Elements 4 to 6 in Group 6; the bottom panel displays the intensity profiles across the vertical lps. (C) Image of the lps from Groups 4–6 of the resolution target captured from the MR headset. (D) The upper panel shows the intensity profiles across the horizontal lps of Elements 4 to 6 in Group 6; the bottom panel shows the intensity profiles across the vertical lps.

We assessed the resolution of the raw images captured by the MR-SLP cameras and the images rendered in the MR headset and compared them with the view directly through the slit lamp eyepieces, using an US Air Force 1951 resolution target as the imaging subject. With a 25× optical magnification, the spatial resolutions measured from the MR-SLP cameras, and the MR headset were found to be identical. The minimum readable line pairs (lps) were identified as element 5 in group 6, which corresponds to 102 lps/mm, with a line width of approximately 9.8μm between two lines (Fig. 3). Three participants, who had a best corrected visual acuity (BCVA) of 20/20, reported that the minimum readable line pairs ranged from element 5 to element 6 (114 lps/mm) when viewed through the slit lamp eyepieces; however, only element 5 was discernible through the MR headset. All participants noted that the lines appeared slightly clearer when viewed through the slit lamp compared to the MR headset.

### Depth perception of MR-SLP

**Fig. 4.**
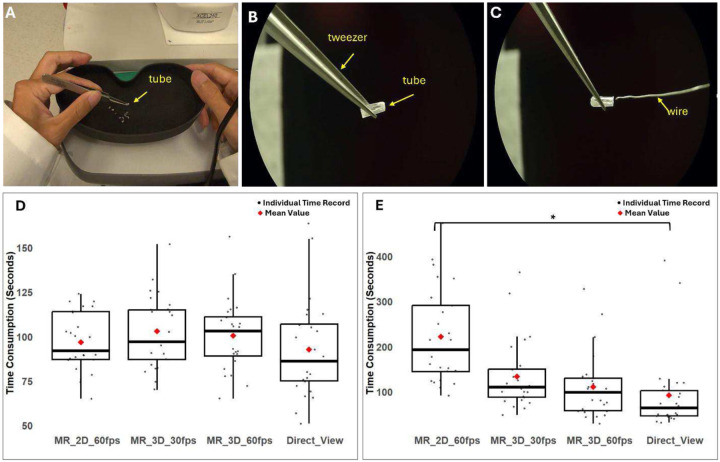
The tube-threading test and results. (A) The participant uses tweezers to grasp a tube, (B) positions the tube into the slit lamp’s field of view until it is clearly visible, and (C) threads a wire into the tube under the slit lamp’s view. (D) Box plot of $T_{\text{task}1}(p,c,i)$ across the four test conditions. (E) Box plot of $T_{\text{thr}}(p,c,i)$ across the four test conditions. Each dark dot represents an individual time record, and the red diamond symbol is the mean value. The asterisk (*) denotes the existence of a statistically significant difference (p <0.05) between two conditions.

To assess the depth perception of the MR-SLP, a tube-threading test was designed based on the concept of bead-threading task^11,12^, a well-known method for estimating fine visuomotor performance mostly effected by depth perception. This tube-threading test, as shown in Fig. 4, consisted of two sequential tasks. In task 1, the participant was asked to pick up each of the ten tubes from a container using a tweezer, place the tube at the focal plane of the slit lamp to be seen clearly, and then return the tube to the container. In task 2, the participant was asked to pick up each tube, thread a wire into it with the visual guidance of the slit lamp and return the tube to the container. The participants were required to complete two tasks five times under each designed condition shown in Table 1. The time taken for each task under each condition was individually recorded, denoted as $T_{\text{task}1}(p,c,i)$ and $T_{\text{task}2}(p,c,i)$, where p is the participant number, c is the condition ID, and i is the repeat.

From a single repeat, the time to thread the tube $T_{\text{thr}}(p,c,i)$ was calculated as

(1) $$T_{\text{thr}}(p,c,i)=T_{\text{task}2}(p,c,i)-T_{\text{task}1}(p,c,i)$$

The mean of $T_{\text{task}1}(p,c,i)$ and $T_{\text{thr}}(p,c,i)$ from the five repeats of a given participant under the same condition were calculated as

(2) $${\overset{‾}{T}}_{\text{task}1}(p,c)=\frac{1}{5}\sum_{i=1}^{5}T_{\text{task}1}(p,c,i),{\overset{‾}{T}}_{\text{thr}}(p,c)=\frac{1}{5}\sum_{i=1}^{5}T_{\text{thr}}(p,c,i)$$

Five people participated in the tube-threading tasks. All the participants successfully completed the test within a reasonable timeframe (< 10 minutes). Their ${\overset{‾}{T}}_{\text{task}1}(p,c)$ and ${\overset{‾}{T}}_{\text{thr}}(p,c)$ records are summarized in Tables 2 and 3, respectively. According to the records, ${\overset{‾}{T}}_{\text{task}1}(p,c)$ was typically the shortest in the direct view condition (c = 4). A Shapiro Wilk test was conducted with ${\overset{‾}{T}}_{\text{task}1}(p,c)$ and ${\overset{‾}{T}}_{\text{thr}}(p,c)$ for each test condition. The test outcome indicated that the assumption of a normal distribution was not applicable in this dataset. Therefore, a non-parametric method, the Kruskal-Wallis test, was used to compare ${\overset{‾}{T}}_{\text{task}1}(p,c)$ and ${\overset{‾}{T}}_{thr}(p,c)$ under different conditions.

As shown in Fig. 4D, the distribution of $T_{\text{task}1}(p,c,i)$ for all four test conditions are similar. This observation was confirmed by the result of the Kruskal-Wallis test, indicating that there were no significant differences among ${\overset{‾}{T}}_{\text{task}1}(p,c)$ for all four conditions (n=20, P=0.77). According to the trend depicted in Fig. 4E, the mean value of ${\overset{‾}{T}}_{thr}(p,c)$ for each condition was the shortest in the direct view condition (c = 4) and gradually increased from test condition 3 (MR 3D 60 FPS) to test condition 2 (MR 3D 30 FPS) and then to test condition 1 (MR 2D 60 FPS). A statistically significant difference in ${\overset{‾}{T}}_{\text{thr}}(p,c)$ was observed among the four test conditions according to the Kruskal-Wallis test (n=20, P=0.037). Subsequent post hoc analysis using Dunn’s test with Bonferroni Correction revealed that only ${\overset{‾}{T}}_{\text{thr}}(p,1)$ and ${\overset{‾}{T}}_{\text{thr}}(p,4)$ are statistically significantly different, meaning statistically significant difference exists only between test condition 1 (MR 2D 60 FPS) and test condition 4 (Direct View) (n=5, P=0.028). This analysis showed that the performance of the threading task has no significant difference between MR headset working on 3D mode and viewing directly through the eyepieces. However, the results also tell us increasing the image resolution and the frame rate will reduce the time taken for the threading task.

### Users experience with MR-SLP

**Fig. 5.**
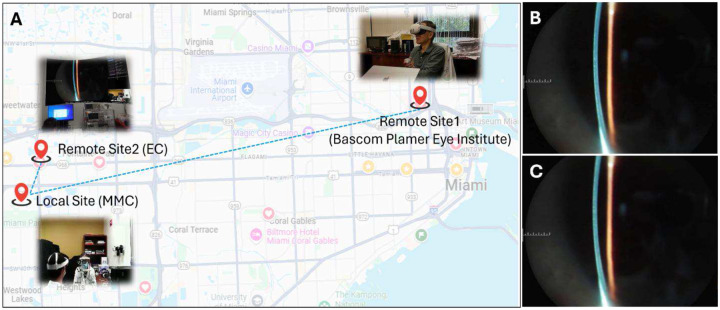
Test of MR-SLP for tele-ophthalmology. (A) The geographical relationship between the local site (FIU main campus) and the remote sites (FIU Engineering Center and Bascom Palmer Eye Institute) involved in assessing image quality and user experience of the MR-SLP. (B) The images obtained directly from the camera of the MR-SLP; (C) The video frame displayed on the remote MR headset at the same time. No noticeable differences were observed between these two images.

A subjective user experience assessment was conducted to evaluate the streaming quality, remote usability, and applications of the MR-SLP in telemedicine. In the assessment trial, as depicted in Fig. 5, the MR-SLP system’s ability to simultaneously transmit high-resolution stereoscopic video to both local and remote MR headsets was successfully demonstrated. At the local site, the operator wearing the MR headset could simultaneously see the patient through the HMD’s see-through function and view the stereoscopic examination images immersed in their real-world environment. Meanwhile, remote users connected via the internet can concentrate on the stereoscopic examination images, offering real-time expert guidance and diagnostic input, promoting effective collaboration over distances.

**Fig. 6.**
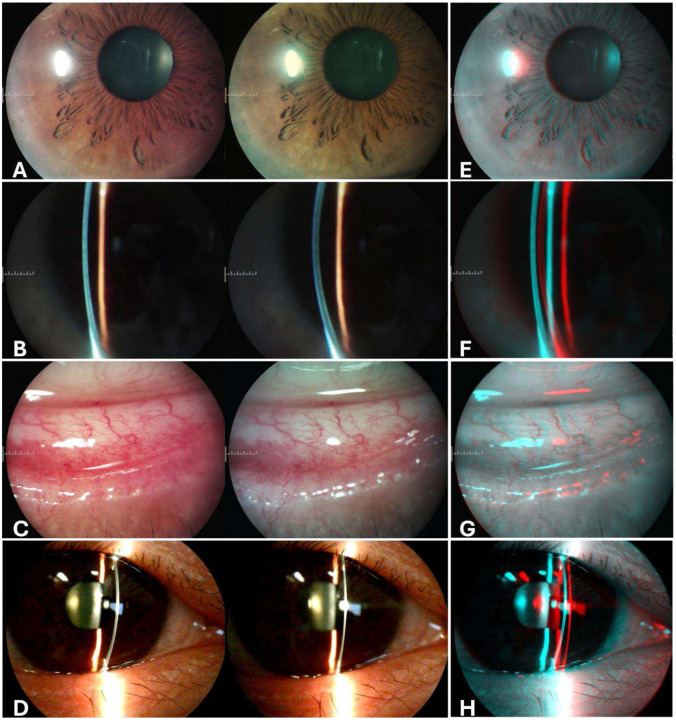
Images of the anterior segment structures captured by MR-SLP locally in SBS format. A: Iris; B: Corneal cross-section (blue stripe); C: Conjunctiva; D: Lens. (E, F, G, H): Images in Red-Cyan stereoscopic format demonstrate the 3D view that can be observed using red, cyan anaglyph 3D glasses.

During the examination of the volunteer’s eye with the MR-SLP, the anatomical details of the anterior segment of the eye were accurately captured and displayed in real time in both local and remote MR headsets (Fig. 6). According to the ophthalmologists participating in this trial, they believed the image quality viewed in their MR headsets closely rivaled those observed through the slit lamp eyepieces. The real-time video streams in this trial also maintained robust depth perception and exhibited low latency at remote sites, with an average round trip time of less than 40 ms and video jitter below 20 ms. This allowed for a highly interactive and collaborative examination by the local and remote ophthalmologists. Both ophthalmologists involved in the trial reported that the MR-SLP system compared favorably to traditional slit lamp viewing and highlighted its potential advantages for clinical practice.

## Discussion

We have tested the performance and validated the feasibility of the MR-SLP for providing stereoscopic slit lamp views of the eye in real time both locally and remotely. The MR-SLP potentially will provide a paradigm shift for slit lamp operations, making it safer for both operators and patients by eliminating the need for close physical proximity. It will improve the efficiency of slit lamp operation training by enabling multiple participants to see real time stereoscopic diagnostic images as if everyone is looking through the eyepieces and performing the diagnosis in person. The remote ophthalmologist wearing the XR headset will have immersive experiences as if operating the slit lamp directly, making 3D tele-ophthalmology possible.

The video quality of the MR-SLP at both local and remote sites can rival that of looking through the eyepiece of a conventional slit lamp. The MR headsets used in this study have a display resolution of approximately 2k per eye while the cameras have a maximum resolution of 4k. The measured spatial resolution was similar (102 lps/mm) for both the images acquired by the MR-SLP cameras and the MR device, suggesting that at the tested magnification (25×) the system’s effective resolution was limited by the slit lamp optics. It is also possible that the MR device’s rendering algorithms enhance the perceived clarity in the region of interest, compensating partially for the lower display pixel density^13^.

Accurate depth perception through the slit lamp is essential for precise clinical diagnosis and surgical procedures^14^. Our tube-threading test confirms that MR-SLP provides depth perception comparable to a traditional slit lamp. However, all the participants stated that the tubes appeared clearer when viewed directly through the eyepieces. We believe this was caused by the effective resolution of the cameras employed in this study during video streaming. The output frame rate was set at 60 FPS only when the camera was operated at a resolution of 720P, which was limited by the camera hardware. This issue can be resolved by using cameras that provide 1080P videos at a frame rate of 60 FPS, which are abundant in today’s market. The results of the tube threading study, shown in Fig 2, also indicate that a higher frame rate leads to a better performance. This improvement may be attributed to the reduction of the motion blur at higher frame rate^15^. Future studies should explore how to best balance maintaining high frame rates with achieving higher resolutions, such as 1080p or 4K, for these dynamic procedural tasks.

System latency is a crucial factor in real-time applications, such as telemedicine, because delays can significantly impact local operator’s performance and diminish the quality of remote interactions. Latency can arise from network transmission, data processing, and display rendering^16^. While acceptable latency thresholds for demanding applications like telesurgery are suggested to be below 100 –200ms^17,18^, platforms utilizing WebRTC architecture can potentially achieve very low media transmission delays^19^. Encouragingly, in our local testing, the participants reported non-noticeable delay during the tube-threading task. Furthermore, remote tests conducted within the same city demonstrated good performance with an average Round Trip Time (RTT) below 40ms. Nevertheless, further evaluation through long-distance remote testing is needed.

With MR-SLP, operators are not limited to a fixed eyepiece position, allowing greater freedom of movement. In other words, the system can provide a position-free solution to operate slit lamps locally and a location-free solution to conduct examination remotely. Additionally, the camera adapter and software implementation are cost-effective and require minimal modification, allowing for versatile integration with most slit lamps and potentially other binocular microscopes.

In the current implementation of the MR-SLP system, Meta Quest 3 is used as the MR headset for both the local and remote locations. Quest 3 offers advantages for biomedicine applications, including high-resolution displays and advanced color passthrough that enable detailed 3D visualization of anatomical structures. Its see-through function and accurate spatial mapping facilitate natural interaction and situational awareness, allowing users to seamlessly integrate virtual content with the real clinical environment. Additionally, Quest 3’s onboard processing power efficiently converts side-by-side images into immersive 3D views, supporting real-time telemedicine and collaborative workflows. However, the headset’s ergonomics and comfort remain suboptimal, particularly during prolonged use. While the display resolution is high, further improvements — such as 4K or higher per eye — would be beneficial for even greater image clarity. We are also investigating alternatives to Quest 3, such as Apple Vision Pro, or AR glasses.

In conclusion, this study successfully demonstrates the capability of an MR-SLP in capturing and transmitting real-time, stereoscopic slit lamp video to MR headsets with low latency and high fidelity, offering depth perception and image quality comparable to direct viewing. This technological integration provides a novel, location-flexible method for slit lamp examination. It could enhance medical image visualization, tele-ophthalmology consultations, and immersive training environments for ophthalmology students and surgeons. While achieving performance close to direct viewing in key metrics, future work should focus on optimizing the resolution and frame rate of the streamed video, quantifying depth perception accuracy, performing long-distance remote testing, and exploring clinical utility across diverse diagnostic and procedural scenarios. The MR-SLP represents the first step toward XR-ophthalmology.

## Methods

### System design

The MR-SLP system developed in this study was built on a commercial slit lamp (XCEL250, Recheit Inc., Italy). Two board-level USB video cameras (ELP-USB16MP01-L75, 1/2.8-inch sensor, maximum resolution: 4656×3496 pixels) were mounted on the left and right eyepieces using custom-designed adapters. These adapters were engineered to achieve optimal optical alignment between the slit lamp eyepieces and the cameras, effectively eliminating artifacts such as vignetting, distortion, and partial image loss. Additionally, the adapters allowed for precise camera adjustments—including image centering, projection distance, and rotation—to ensure stereoscopic depth perception in the MR headset consistent with the original slit lamp optics. For practical use, the adapters incorporated a locking mechanism to maintain mechanical stability during routine operations and featured a user-friendly design for quick and easy attachment and detachment.

**Fig. 7.**
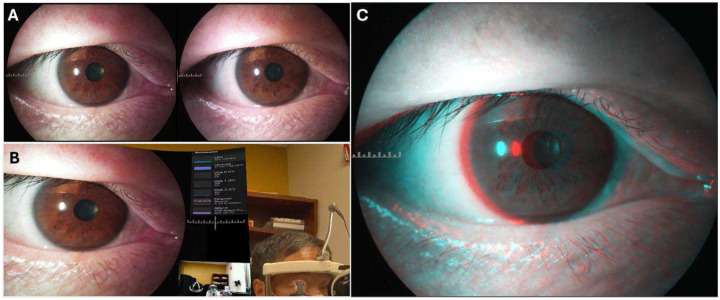
Imaging procedures from camera to the HMD rendered display. (A) Example images of a subject’s eye captured with the MR-SLP cameras in the SBS format. (B) The view of the local operator through their MR headset. The embedded window on the left displays the stereoscopic view (rendered from the SBS video) of the subject’s eye. The subject and the environment were seen with the see-through function of the MR headset. (C) An image in Red-Cyan stereoscopic format demonstrates the 3D view as an example that can be observed using red, cyan anaglyph 3D glasses.

The videos captured by the two cameras were first streamed via USB to a local computer, where they were processed in real time using Open Broadcaster Software (OBS) Studio (64-bit, v31.0), an open-source video streaming application. The processed videos were formatted into side-by-side (SBS) mode (Fig. 7A), a widely used format for conveying stereoscopic information in image processing^20^. Subsequently, the SBS video was transmitted to local and remote WebRTC servers using the WebRTC HTTP Ingest Protocol (WHIP), with H.264 encoding at a target bitrate of approximately 10 Mbps and an output resolution of either 1080p or 720p. For local streaming, a free WebRTC server (OSSRS/SRS v6.0 from Docker Hub) was deployed on a PC, while remote streaming was facilitated by a commercial WebRTC service provided by dolby.io over the internet. The processed streams were broadcasted wirelessly to MR headsets using the WebRTC HTTP Egress Protocol (WHEP). In this study, Meta Quest 3 was used as the MR device for both local and remote sites, with its built-in video player rendering the SBS video into a stereoscopic view for immersive visualization (Fig. 7B).

### Spatial resolution evaluation

A US Air Force 1951 resolution target (Edmund Optics Inc., Barrington, NJ) was used to evaluate the spatial resolution of MR-SLP. The target was placed at the imaging plane of the MR-SLP and imaged at 25× optical magnification. Raw images of the resolution target were captured using the MR-SLP’s USB cameras with the built-in camera app on Microsoft Windows 11, at a resolution of 4,656×3,496 pixels, which is the cameras’ maximum resolution. Images displayed in the MR headset were captured through real-time video streaming, using the built-in snapshot tool of the Meta Quest 3.

Intensity profiles were generated across the line pairs for selected group–element combinations from the acquired resolution target images using a custom MATLAB script. The minimum resolvable line-pairs were determined according to the Rayleigh Criterion, wherein the central maximum of one pattern coincides with the first minimum of another. Additionally, a subjective evaluation was conducted in which participants compared the minimum readable line-pairs observed directly through the eyepieces with those visible in the images captured via camera.

### Tube-threading test for evaluating depth perception of MR-SLP

A high-precision tweezer (PL-30, Fisher), primarily for microscopy applications, was used to grab the tubes. The tubes used in this test were made from micro pipette tips. Their outer diameter ranged from 0.8 mm to 1.6 mm, and their inner diameter ranged from 0.4 mm to 1.2 mm. The diameter of the wire used in this test is 0.2 mm. The small size of these tubes and the wire, combined with 10× optical magnification of the slit lamp, required a precise depth perception to complete the designed tasks.

Five individuals were recruited to participate in this test. Each participant had normal visual function, with a best corrected visual acuity (BCVA) of 20/20 and a minimum stereoacuity of approximately 40 arc seconds, assessed using R.D.S. random-dot stereograms^21^. The participants were required to complete the test five times under each designed condition. $T_{\text{task}1}(p,c,i)$ and $T_{\text{task}2}(p,c,i)$ taken for each task under each condition was individually recorded. ${\overset{‾}{T}}_{\text{task}1}(p,c)$ and ${\overset{‾}{T}}_{\text{thr}}(p,c)$ for all four conditions were collectively analyzed using statistical methods to identify the effects of the test conditions on participants’ performance.

### User experience assessment

The user experience assessment involved five participants assigned to different roles across three locations in Miami. The local site was at the main campus of Florida International University (FIU), where an ophthalmologist with around 10 years of clinical experience operated the MR-SLP and performed test examinations on a volunteer with normal visual condition. Stereoscopic slit lamp video was captured and streamed in real time at an output resolution of 1080p. Simultaneously, a second ophthalmologist with over 20 years of clinical experience watched the stereoscopic video from the MR-SLP using his MR headset at the first remote site - Bascom Palmer Eye Institute at the University of Miami Miller School of Medicine. He communicated in real-time with the MR-SLP operator at the local site, providing expert guidance and diagnostic opinions. In addition, a senior scientist at the first remote site also observed the examination procedure through his MR headset for technical assistance. Lastly, a senior scientist at the second remote site, the FIU Engineering Center, observed the process using his MR headset. All local and remote participants verbally assessed the system’s technical performance, including image quality and streaming stability. The latency, particularly with RTT, was provided by the remote WebRTC platform.

## Figures and Tables

**Table 1.** Test conditions utilized in the tube-threading evaluation

ID (c)	Conditions	Description
1	MR-SLP, 2D 60 FPS	Non-stereoscopic stream*, output resolution: 720p, frame rate: 60 FPS, with MR headset.
2	MR-SLP, 3D 30 FPS	Stereoscopic stream, output resolution: 720p, frame rate 30 FPS, with MR headset.
3	MR-SLP, 3D 60 FPS	Stereoscopic stream, output resolution: 720p, frame rate: 60 FPS, with MR headset.
4	Conventional Slit Lamp	Direct view through the eyepieces of the slit lamp, without MR headset.

* Non-stereoscopic stream was created by converting the video from a single MR-SLP camera into the SBS format.

**Table 2.** ${\overset{‾}{T}}_{\text{task}1}(p,c)$ of all participants under all four test conditions.

*Participant (p)*	*Conditions (c)*			
	1	2	3	4
	MR 2D 60fps	MR 3D 30fps	MR 3D 60fps	Direct View
***1***	77 ± 9	92 ± 7	84 ± 18	63 ± 9
***2***	107 ± 12	126 ± 15	111 ± 7	116 ± 29
***3***	118 ± 4	120 ± 8	124 ± 22	107 ± 9
***4***	91 ± 5	93 ± 8	91 ± 2	75 ± 5
***5***	91 ± 11	85 ± 17	91 ± 19	103 ± 31

Time was recorded in seconds, and each number represents the mean ± standard deviation (SD) from the five repeats.

**Table 3.** ${\overset{‾}{T}}_{\text{thr}}(p,c)$ of all participants under all four test conditions.

*Participant (p)*	*Conditions (c)*			
	1	2	3	4
	MR 2D 60fps	MR 3D 30fps	MR 3D 60fps	Direct View
***1***	241 ± 100	138 ± 77	153 ± 124	68 ± 29
***2***	317 ± 119	133 ± 39	110 ± 20	76 ± 38
***3***	131 ± 26	92 ± 14	75 ± 30	57 ± 18
***4***	175 ± 56	93 ± 37	69 ± 25	48 ± 10
***5***	249 ± 113	213 ± 120	149 ± 81	215 ± 139

Time was recorded in seconds, and each number represents the mean ± standard deviation (SD) from the five repeats.
